# Applied mathematical modelling to inform national malaria policies, strategies and operations in Tanzania

**DOI:** 10.1186/s12936-020-03173-0

**Published:** 2020-03-02

**Authors:** Manuela Runge, Fabrizio Molteni, Renata Mandike, Robert W. Snow, Christian Lengeler, Ally Mohamed, Emilie Pothin

**Affiliations:** 1 Swiss Tropical and Public Health Institute, Basel, Switzerland; 2 University of Basel, Basel, Switzerland; 3 National Malaria Control Programme, Ministry of Health Community Development Gender Elderly and Children, Dodoma, Tanzania; 4 Centre for Tropical Medicine and Global Health, Nuffield Department of Clinical Medicine, University of Oxford, Oxford, OX3 7LJ UK; 5 Population Health Unit, Kenya Medical Research Institute-Wellcome Trust Research Programme, Nairobi, Kenya; 6 CHAI, Clinton Health Access Initative, Boston, USA

**Keywords:** Malaria, Strategic planning, Mathematical modelling, Tanzania, Stratification

## Abstract

**Background:**

More than ever, it is crucial to make the best use of existing country data, and analytical tools for developing malaria control strategies as the heterogeneity in malaria risk within countries is increasing, and the available malaria control tools are expanding while large funding gaps exist. Global and local policymakers, as well as funders, increasingly recognize the value of mathematical modelling as a strategic tool to support decision making. This case study article describes the long-term use of modelling in close collaboration with the National Malaria Control Programme (NMCP) in Tanzania, the challenges encountered and lessons learned.

**Case description:**

In Tanzania, a recent rebound in prevalence led to the revision of the national malaria strategic plan with interventions targeted to the malaria risk at the sub-regional level. As part of the revision, a mathematical malaria modelling framework for setting specific predictions was developed and used between 2016 and 2019 to (1) reproduce setting specific historical malaria trends, and (2) to simulate in silico the impact of future interventions. Throughout the project, multiple stakeholder workshops were attended and the use of mathematical modelling interactively discussed.

**Evaluation:**

In Tanzania, the model application created an interdisciplinary and multisectoral dialogue platform between modellers, NMCP and partners and contributed to the revision of the national malaria strategic plan by simulating strategies suggested by the NMCP. The uptake of the modelling outputs and sustained interest by the NMCP were critically associated with following factors: (1) effective sensitization to the NMCP, (2) regular and intense communication, (3) invitation for the modellers to participate in the strategic plan process, and (4) model application tailored to the local context.

**Conclusion:**

Empirical data analysis and its use for strategic thinking remain the cornerstone for evidence-based decision-making. Mathematical impact modelling can support the process both by unifying all stakeholders in one strategic process and by adding new key evidence required for optimized decision-making. However, without a long-standing partnership, it will be much more challenging to sensibilize programmes to the usefulness and sustained use of modelling and local resources within the programme or collaborating research institutions need to be mobilized.

## Background

### Why use modelling for strategic planning?

The concept of using mathematical modelling for strategic planning of infectious disease control is not new [1–3]. Multiple examples exist for a wide range of infectious diseases [4–8] and specifically for malaria [2, 9–12]. Mathematical modelling uses available information to generate data-driven simulations of transmission dynamics and control for specified settings [2, 9, 13, 14]. The model predictions can quantify with some uncertainty the expectations of the impact of interventions for different areas. The exploration of alternative scenarios aids in decision-making and facilitates a more strategic approach in the selection of interventions [15–18]. More than ever, it is crucial to make the best use of existing country data and analytical tools [19] because: (1) there is an increasing complexity with the expanding available malaria control tools as a result of effective research and development, (2) the local epidemiology is becoming more heterogeneous as a result of massive ongoing control efforts, and (3) resources, especially funding, are not increasing. Hence, global and local policymakers, as well as funders, increasingly recognize the value of mathematical modelling as a strategic tool to support decision-making [1, 12] (Table 1). In addition, growing stakeholder coordination and the need to use evidence will lead to more strategic questions about priorities and combination of interventions. In parallel, more and better quality data become available in endemic settings, enhancing the value of modelling [2].

**Table 1:** Value of modelling for strategic planning of malaria control interventions

Additional layer of information collating all available evidence to disentangle key determinants,predict expected impact and identify knowledge or data gaps
Generation of hypotheses and guidance of decisions by comparing scenarios that might not necessarily have been evaluated on the ground
Establishment of an interdisciplinary platform for structured discussions on strategies
Assessment of technical feasibility to achieve specific goals that can be useful in the context of strategic plan updates, funding applications, prioritization of interventions, and operational planning

These developments clearly call for a more sustainable and in-depth relationship between modellers, NMCP managers and donors. Given the historical difficulty of linking modelling and strategic planning, intensified technical support, closer interactions and capacity building within-country NMCPs are required. This case study presents such close collaboration between modellers, donors and the NMCP managers, providing a unique and effective example of modelling for strategic planning.

### Geographic specific malaria modelling

Mathematical models have been applied for various countries at varying resolutions, examples include sub-Saharan African (SSA) countries [15, 20–22], Ghana [23, 24], Kenya [25, 26], Mozambique [27, 28], Nigeria [16, 17, 29], Uganda [30], South Africa [31], Zambia [32–35], and the Asia–Pacific Region [36, 37]. In those examples, modelling was used to investigate relevant transmission dynamics, intervention effectiveness or for stratification. While sometimes useful for global policy writing, there have been fewer examples where mathematical modelling has been applied in a country at the required operational unit and accompanied with a national policy dialogue. Exceptions are Zambia [35], Ghana [24], South Africa [31], Cambodia and Thailand (Mahidol Oxford Research Unit (MORU)), Mozambique, Sri Lanka, Phillippines, Benin (Swiss Tropical and Public Health (Swiss TPH)). In Kenya, Tanzania and Uganda, a decision support tool has been developed in communication with local stakeholders, to link research and policy for “guiding the selection of more effective, evidence-based control strategies” [14, 38]; however, no country-wide application could be found.

### Country application Mainland Tanzania

In 2016, a team of modellers from Swiss TPH were invited by the Global Fund to Fight AIDS Tuberculosis and Malaria (GFATM) to provide support to the Tanzanian NMCP for preparing the upcoming funding request [39]. After this initial undertaking ended in early 2017, the NMCP and the Swiss TPH team suggested to continue modelling which then could be made an intrinsic part of the on-going planning processes of the NMCP. The sections below describe the non-technical process of applying mathematical modelling, its added value, challenges and lessons learned. The development of the modelling approach is described in [40] and the results of modelling application are included in the Supplementary Midterm Malaria Strategic Plan 2018–2020 [41].

### Partnerships and collaborations

The Swiss TPH has a long-established relationship with the NMCP in Tanzania. In 2002, the Swiss Agency for Development and Cooperation (SDC) launched the NETCELL project to provide technical and strategic support to the NMCP, with the Swiss TPH as implementing partner [42]. Since its launch, NETCELL contributed to the strengthening of the NMCPs capacities to plan, coordinate, and implement malaria control interventions, in particular insecticide-treated bed nets (ITNs) [43, 44]. The NETCELL team collaborates with the Ministry of Health, Community Development, Gender, Elderly and Children (MoHCDGEC), the President’s Office, Regional Administration and Local Government (PO-RALG), UK Department for International Development (DfID), United States Agency for International Development (USAID), Worldbank, GFATM, among others [42]. The Swiss TPH modelling team closely worked with the NETCELL team, which in turn facilitated the interactions between the modellers and the NMCP programme members. The NETCELL project has recently been renewed under the financing of the SDC and has many more years to provide continuous support to the MoHCDGEC. Another important regional partner was the KEMRI-Wellcome Trust Programme, who managed DFID funded projects (INFORM and LINK) [45] to provide spatial epidemiological analytical support using nationally available malaria data for subnational decision making in Tanzania and other NMCPs across Africa [46, 47].

### National malaria strategic planning

**Fig. 1:**
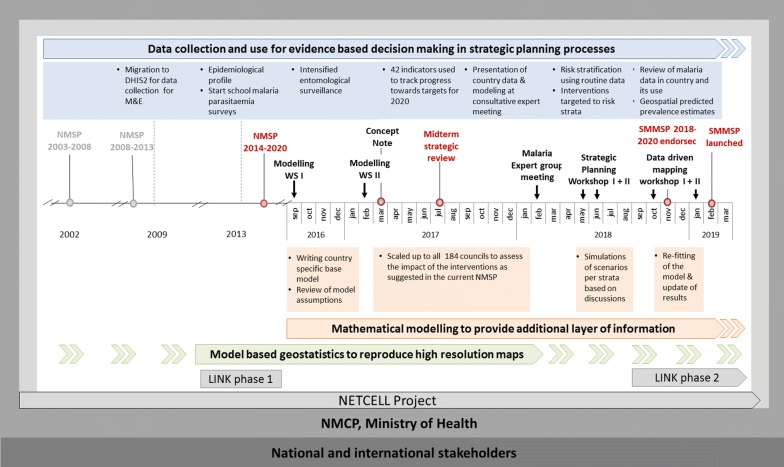
Timeline of events leading to the supplementary malaria midterm strategic plan 2018–2020. A summary of the attended meetings including modelling is provided in Additional file 1

Strategic planning in Tanzania is based on a strong malaria monitoring and surveillance system, including high-quality district health information system (DHIS2) data [48], entomological surveillance [49], resistance monitoring [50], demographic and health surveys, and malaria indicator surveys [51–55]. Since 2014, nationwide annual school malaria parasitaemia surveys also bring high-quality and high-resolution cross-sectional data to the NMCP database [56]. The Tanzanian epidemiological data show nowadays a highly heterogeneous malaria transmission and burden throughout the country [51–56]. The National Malaria Strategic Plan (NMSP) for 2015–2020 acknowledged that diversity of malaria transmission and disease burden within the borders of Mainland Tanzania, but largely adopted a uniform approach to disease management and prevention nationwide [57]. An increase in national average prevalence from 9.5 to 14.8% between 2012 and 2015–16 [52, 53], led to the questions of whether the current NMSP would technically be feasible to achieve the national target, of a prevalence of less than one per cent in 2020. In line with this question arose the issue of optimizing intervention mixes according to endemicity and key epidemiological parameters. As a result, a decision was made by the NMCP to work on a supplementary malaria midterm strategic plan aiming at optimal intervention mixes in different epidemiological strata to ensure optimal impact for available resources [41]. A timeline describing the events leading to the supplementary malaria midterm strategic plan is shown in Fig. 1.

In 2016, two workshops were held in Dar es Salaam to introduce the concepts of modelling, to assess available data sources and data owners, and to discuss input parameters and model assumptions. Following these two workshops, an extended phase was used for the model calibration. In 2017, the results of the initial models were fed into a midterm-review of the strategy, which concluded that the national prevalence target of less than one per cent by 2020 would not be achievable. Indeed, the modelling results suggested that the current NMSP objective could not be achieved unless a much more aggressive intervention mix was put in place. Unfortunately, that was neither operationally feasible nor financially doable. At a malaria expert meeting held in February 2018, with national and international stakeholders, the modelling results were presented alongside with the empirical view of the NMCP on the country context. At that meeting, it was decided to (1) gather all available data for risk stratification at council level, and (2) put together a more detailed plan for improved targeting of interventions at council level.

In May 2018, the modelling team was invited alongside NMCP staff and the NETCELL team to join a strategic planning workshop. During that meeting, the NMCP and stakeholders stratified the councils according to malaria risk (Thawer et al. pers. commun.) and discussed the allocation of appropriate interventions targeted to the strata. The previously calibrated transmission model (using OpenMalaria) was then used interactively during the work session by simulating requested alternative scenarios and directly answering questions from the country programme. Finally, selected outputs of the model were included as an additional set of evidence in the revision of the strategic plan launched in February 2019 [41].

### Added value

Mathematical modelling allowed primarily a technical assessment of the national malaria targets. Once calibrated, predictions of the likely impact of current and potential future interventions at council level could be provided. Beyond the simulation results, the process in itself was useful to inform policy. Modelling did not only use and process quantitative data, but also expert opinions, programme experiences, and local knowledge. Together, these created a platform for an in-depth interdisciplinary dialogue. Presenting model assumptions and the comparisons of the predicted *versus* expected impact triggered controversial as well as constructive comments. Controversial or unexpected predictions led to a critical review of the data, model structure, assumptions made, as well as the planned intervention scenarios. The ongoing engagement between modellers and practitioners enabled knowledge transfer and established a long-term interest in modelling. The former one was demonstrated by a developed critical but more appreciative view which replaced an initial misconception about modelling (i.e. “*why to use modelling when you have data”* changed to *“why is the model different from the data, and how would the predictions change if…*”). The interaction and close collaboration were also of great benefit to the modellers, as the local knowledge and data were invaluable for model improvements leading to more context-specific modelling.

Moreover, statistical modelling and traditional descriptive analyses were performed to describe temporal and spatial trends based on empirical data and not on dynamics of malaria transmission as the mathematical model used has. Indeed, dynamic transmission model use available data to inform parameters to simulate malaria transmission and burden based on an understanding of the transmission dynamics, while statistical models only infer relationships based on collected data, without necessarily understanding the system. Discussions with partners on data for input parameters and major model assumptions were highly relevant to understand and inform the main drivers of malaria transmission. As a direct illustration, the prevalence predictions from the geospatial model provided by KEMRI-WT were discussed between partners including the NMCP and decided to be used to calibrate the transmission model for council prevalence.

### Challenges

A number of challenges affected the accuracy of the modelling outputs and timeliness of the project. First, there was no previous experience for country modelling available at that level of detail that could have guided the process and the type of required outputs. Second, methodological challenges led to extended times for model calibration and complicated uncertainty estimates around the predictions [40]. Uncertainty resides in model predictions. This uncertainty can be due to data quality and accuracy for model parameters, or due to model structure and random variability. The advantage of a simulation model would be to assess the impact of this uncertainty on the predictions. However, given the fact that this framework is representing each council of the entire country, the computational power becomes challenging. As a result, assessment of uncertainty was kept to its minimal, only accounting for random variability by using multiple runs for the historical simulation period, and accounting for uncertainty in transmission intensity by fitting a range of transmission intensities to prevalence estimates. Third, gaps in communication and understanding slowed down the process, requiring much more frequent and in-depth engagement between the modelling team and NMCP staff than had been anticipated. Fourth, challenges also included the busy schedule of the NMCP staff, as well as tight deadlines expected by external donors. Moreover, building capacity within the NMCP without a dedicated modelling person within the NMCP or at least within a local institution was challenging and the NETCELL advisory team was invaluble to bridge that gap. However, in order to sustain the modelling support on the long term, the analytical capacities within the NMCP need further strengthening i.e. through additional personnel with quantitative skills, training and increasing experience as the modelling application continues. The first phase of the project has been to set up a framework and ensure engagement with and usefulness for the programme, the second phase will be to transfer knowledge by training in-country modellers. Lastly, it took time to build trust between all partners, to be able to understand the strengths and limitations of the models. The main key challenges and their implications are summarised in Table 2.

**Table 2:** Challenges and their potential implications for a productive interaction between modelling teams and NMCP staff

Challenges	Implications
No previous experience with country modelling at that level of detail, hence need to create process Short timelines especially by external donors Insufficient time of NMCP staff for required activities Delays by NMCP in data sharing Delays by modellers in getting a clear understanding of the available data in order to increase accuracy of model parameters based on the available data Use of a complex transmission model and long processing time of simulations	Need for NMCP to invest required time in interactions—depending critical on NMCP understanding value of modelling and the process of interactions Prolonged time for model set up and calibration Delays in modelling deliverables and missed opportunities to inform key decisions Additional resources needed to extend the project period in order to adequately improve technical aspect and standardize processes to provide timely deliverables
Low spatial resolution for most indicators and temporal data gaps Use of most of the available data to inform the model while reducing the number of assumptions made Inclusion of model complexities and uncertainties while simplifying the model to shorten simulation time	Increased uncertainty in model parameters and predictions and impossibility to use model predictions at a higher resolution Undermining of model usefulness and credibility and potential reluctance towards future modelling applications
Maintaining communication between in-country visits between modelling team and NMCP Need to use a simplified language without leaving out relevant technical details Transparency on model limitations and uncertainty without undermining perceived modelling value Negative perception towards modelling by some stakeholders Misunderstanding the role of modelling as a replacement instead of an addition to data	Loss of interest in modelling process that could potentially lead to a negative perception of its use. Constant need to highlight the practical contribution made by models and the process of interaction with NMCP
Conflicting deadlines for activities at the NMCP level Difficulty to find in-country personnel to train for taking over the methodology Project funding with a focus on short term deliverables rather than long-term support	Dependency on external modeller and temporary project funds that prevent sustained effort and gains of the initiative Missed opportunity for improvements and refinements to shape the model into a truly setting specific tool and use of its maximum potential

### Key components for successful modelling use in strategic planning

Once modelling activities were understood and adopted by the NMCP (and not perceived only as an academic exercise), the modelling process was used systematically as a way to think about the data. Furthermore, model strengths and limitations became better understood by the NMCP and partners, making the entire effort more productive. Ultimately, the whole process fed into the strategic planning process through interactive presentations and discussions. This exchange allowed for an additional layer of thoughts and interpretation and was found to be essential for the model to be meaningful and appropriate at the end Additional file 2. To achieve this, multiple interactions, workshops and demonstration of the model were required. The NETCELL team made up of technical experts understanding both programme constraints, and the basics of modelling facilitated the communication by ‘translating’ between technical language to programmatic language. The NETCELL team also ensured continuity in the process, especially in-between visits by the modelling team. Their country-specific knowledge and resources were invaluable for many aspects of the modelling. A summary of the critical elements for success identified throughout the process is provided in Table 3.

**Table 3:** Key components for successful modelling use in strategic planning at country level

Component	Recommendation	Relevance
Context		
Ownership	The modelling should be led by the Ministry of Health through the NMCP while including all other key stakeholders	Coordination of partners and activities centred around country needs and country-specific questions
Aim & purpose	The aim of the modelling application should be clear to all stakeholders involved with defined deliverables	Establishment of transparent and shared expectations of modelling output and impact
Data sharing & accessibility	Relevant data from local research or governmental institutions should be made available to programme managers and modelling team	Reinforcement of country-ownership and enhanced use of data
Data quantity & quality	Data quality and suitability to inform the models need to be assessed, and if necessary, proper adjustments should be made, in consultation with the programme	Improvement of model accuracy and usefulness of predictions
Process		
Timeliness	Timelines need to be set by the programme and need to be sufficient for completion of programmatic as well as modelling tasks	Feasibility of timely deliverables for a successful and efficient strategic planning process
Consistency	A systematic workflow should be developed and consistently be used throughout the project	Reproducibility of modelling results facilitates potential evaluation of applied modelling
Integration	The outputs from programme activities should feed into the modelling process, which in turn should inform the next programmatic activity	Utilization of modelling results by the programme and prevention of unnecessary additional modelling iterations
Monitoring	The modelling outputs should be compared to the parallel activities at the NMCP	Usefulness of modelling targeted to relevant and current country needs in consideration of latest available data
Communication		
Dissemination & Discussion	Modelling process and results should be presented to relevant stakeholders and at the end, final reports and documentation should be made available	Provision of a discussion platform for exchange and knowledge transfer between partners, essential for impactful application of modelling
Engagement, commitment & responsibility	All parties involved should actively participate in the discussions and maintain constant commitment	Opportunity of achieving highest benefit for all partners involved
Understanding	Knowledge transfer (in all directions), and capacity building should be a fixed part of the modelling	Growths of mutual understanding and capacity despite substantial differences in disciplines and technical level between stakeholders
Transparency	The strengths and limitations of modelling need to be transparent	Consideration of modelling as a thinking tool with sensible interpretation of results
Modelling		
Parameterization & calibration	Available data should be used to identify and inform setting specific model parameter and the calibration methodology should account for the historical trends in malaria	Simulation of data-driven impact predictions specific to local settings
Validation	The predictions need to be compared with data not included in the modelling, especially when developing or using new models and parameterizations	Alignment between modelled and observed data earns credibility, whereas discrepancies can be helpful for the identification of knowledge gaps or model improvements
Complexity	The model complexity should be appropriate for the questions asked (“as complex as necessary but as simple as possible”)	Reduction of computational efforts and simplified interpretation of modelling results
Flexibility	The modelling workflow needs to be flexible enough to be able to respond to current country needs and questions as they come up	Prevention of unnecessary modelling iterations and strengthening the potential of modelling as a routine tool integrated into strategic planning processes

## Discussion

The Global Technical Strategy for malaria [19] and its more recent adaptations under the High Burden High Impact (HBHI) initiative [58] emphasize the need to target control strategies. Ultimately, it aims to ensure that future policies are evidence-based and promote country-led and data-driven decision-making [58]. This publication described a unique example of an iterative modelling process resulting from a close collaboration between the NMCP in Tanzania, a modelling team at the Swiss TPH and other stakeholders. Similar experiences and challenges were identified previously in health policy and decision-making research [14, 59–61].

Close cooperation and on-going communication are crucial to prevent on the one hand the risk of overconfidence in model predictions [62], or scepticism from control programme staff leading to a lack of uptake of model outputs. In the presented application, the comparison of alternative scenarios in multiple epidemiological settings provided qualitative guidance. Already described by MacKenzie in 1998, modelling should be used as a “thinking tool” rather than as a “future machine” [62].

In modelling, there is a well-known trade-off between accuracy and simplification, and the acceptable level of the accuracy is defined by the purpose of the model (e.g. operational planning, high-level policy recommendation, advocacy and resource mobilization, or academic exercises). As the interactions between the modelling and NMCP evolve, it will become feasible to make more nuanced use of the data and to broaden the scope of the optimization, while propagating uncertainties throughout the analysis. For instance, council level targets, varying target coverages [20, 21] and sequential introduction of interventions [22] might be considered and more seeds or model variants added to also account for uncertainties in model structure and random variation. The importance of uncertainty when using modelling for decision-making has been addressed in detail elsewhere [63, 64].

It is also essential to set realistic targets and expectations on what modelling can and cannot deliver [6] in a given timeframe. The outputs of the process, described in [40], did ultimately not inform the 2017 concept note for the GFATM application as was initially foreseen. Neither the model outputs nor the NMCP were ready for that exercise because of tight deadlines and additional time required for the model calibration. Had communication been stopped, it could have led to suboptimal utilization of modelling. The long-term process however, was only possible with the dedication of all participants and the steady country support. Without a long-standing partnership, it will be much more challenging to sensibilize programmes to the usefulness and sustained use of modelling, local resources within the programme or collaborating research institutions need to be mobilized.

Modelling received appreciation when it was used for impact predictions of the intervention stratification selected by the programme. This emphasizes the necessity to establish shared ownership of all processes despite knowledge asymmetry, to facilitate the country-led use of modelling. In our application, a country-led use of modelling was achieved with open discussions on data and model uncertainties, with constant raising of questions for the model to answer. Through this first phase of engaging with the Tanzanian NMCP the modellers have raised awareness not only to the NMCP and partners in country themselves but also to a broader community, promoting the need for review of data and benefit of modelling to predict impact of intervention and support decision making processes.

The varying understanding of modelling usefulness by the NMCP and partners and the inability to know what decisions would have been taken in absence of modelling, highlight the difficulty to evaluate impact of modelling in the decision-making process.

Modelling guidelines for country application have been recently published for tuberculosis [65], but no such guidelines exist yet for malaria. The malERA consultative group provides a modelling research agenda [12, 66], and the use of modelling for malaria control and elimination strategies has been described by WHO and partners [1]. However, they do not include practical guidance on how to use modelling collaboratively to make best use of local data for strategic planning at country level. Such guidelines would also facilitate the comparison of multiple models applied for the same questions within and between countries. The example presented here provides valuable challenges and lessons learned and reinforces the urgency of such guidelines in malaria.

## Conclusions

Modelling provides a platform unifying empirical and simulated outputs, where policymakers, technical experts and other stakeholders can discuss and then agree on what constitutes an optimal national malaria control plan. Such discussions need to consider many parameters and priorities and hence must result from constant interactions between programme managers and modellers. In addition, all other national stakeholders including donors, academics and technical/implementation agencies are encouraged to participate in this process. Empirical data analysis and its use for strategic thinking remain the cornerstone for evidence-based decision making. Mathematical impact modelling can then support the process both by unifying all stakeholders in one strategic process and by adding new key evidence required for optimized decision-making. Given that most malaria-endemic countries (1) have now a high level of epidemiological heterogeneity [67] and (2) that all countries are facing a rapidly increasing number of technical and strategic options, it follows that many could benefit from process similar to the one described here. To support this, minimal essential guidelines for country modelling are now urgently needed for improved evidence-based national and local malaria control planning, implementation and evaluation. Local consortia made up by NMCPs, donors and research institutions need then to be established to carry out strategic planning processes. Not only will this allow for faster progress in malaria control impact at a given level of funding, but it represents an essential step for coming close to the goal of finally eliminating malaria.

## Supplementary information

**Additional file 1.** Details of the main interactive activities between 2016 and 2018

**Additional file 2.** Iterative process between modelling and in-country discussions

## Data Availability

Not applicable.

## Abbreviations

DHIS2: Demographic health information system
DHS: Demographic household survey
GFATM: Global Fund to Fight Tuberculosis, Aids and Malaria
HBHI: High Burden High Impact
KEMRI-WT: Kenyan Medical Research Institute, Wellcome Trust
MIS: Malaria Indicator Survey
MORU: Mahidol Oxford Tropical Medicine Research Unit
NBS: National Bureau of Statistics
NIMR: National Institute for Medical Research
NMCP: National Malaria Control Programme
NMSP: National Malaria Strategic Plan
SDC: Swiss Agency for Development and Cooperation
SSA: Sub-Saharan Africa
SMMSP: Supplementary malaria midterm strategic plan
Swiss TPH: Swiss Tropical and Public Health Institute
WHO: World Health Organization
WHO AFRO: World Health Organization African Region
