# Supplementary material for: Applied mathematical modelling to inform national malaria policies, strategies and operations in Tanzania
Source: Malar J. 2020 Mar 2;19:101. doi: 10.1186/s12936-020-03173-0 (PMC7053121; doi:10.1186/s12936-020-03173-0)
Supplement: Supplementary file 1 — Additional file 1. Details of the main interactive activities between 2016 and 2018 [file 12936_2020_3173_MOESM1_ESM.docx]

**Additional Table 1:** Details of the main interactive activities between 2016 and 2018.

|  | Modelling Workshop (#1) | Modelling Workshop (#2) | Consultative Experts  Malaria meeting | Malaria Strategic Plan  Review 1+2 workshops | Tanzania M&E Mapping workshop I + II |
| --- | --- | --- | --- | --- | --- |
| **Organiser** | Swiss TPH and GFTAM | Swiss TPH and GFTAM | NMCP | NMCP | NMCP+ KEMRI Nairobi |
| **Date** | 27 - 28 Oct.  2016 | 22 Mar.  2017 | 26 -27 Feb.  2018 | 14-19 May  28-2 Jun.  2018 | 3 -7 Sep.  2018 |
| **Participants** | ~30 (Day 1) ~15 (Day 2) | ~20 | ~30 | ~20-30 | ~15 |
| **Institutions** | NMCP, NIMR, IHI, NBS, UDSM, WHO , PMI, PATH, PSI, RTI, VectorWorks, LSTM, GFATM, CHAI, Swiss TPH | NMCP, NIMR, IHI, VectorWorks, CHAI, Swiss TPH | MoHCDGEC (CMO), NMCP, NIMR, IHI, PMI, Global lFund, WHO, WHO Afro, VectorWorks, Swiss TPH, KEMRI WT | NMCP, NIMR, IHI, MoHCDGEC, WHO, GFATM, PMI, ALMA, CHAI, Swiss TPH | NMCP, NIMR, IHI, NBS, TMA, UDSM, PMI, KEMRI WT, Swiss TPH |
| **Objectives** | - To introduce modelling concepts to key stakeholders, - To identify key questions to be addressed by the model application, - To discuss expected outcomes - To identify data to be collated & data owner. | - To present preliminary results on calibration and impact of interventions in 8 districts, - To discuss feedback and agree on next steps. | - To critically review, analyse and discuss the elimination agenda in Tanzania, - To deliberate on effective approaches that will help Tanzania to achieve malaria elimination, - To formulate recommendations and experts resolutions. | - To realign the NMSP 2015 - 2020 to current epidemiological and implementation achievements, - To develop and define a pragmatic stratification, - To identify appropriate and tailored intervention packages according to needs, - To model impact and costs, - To redefine the strategic outline and the implementation framework. | - To generate a new prevalence risk map to improve malaria stratification and plan and implement targeted malaria control interventions, - To update modelling results after fitting to new prevalence estimates per district. |
| **Modelling outputs** | - Preparation of modelling, - List of available and data sourced and contacts, - Refinement of modelling questions, - Selection of eight pilot districts for testing methodology. | - Impact of interventions in eight pilot districts, - Comparison of predicted and observed prevalence in 2016, - Workflow to reproduce historical trend and future predictions Collated database. | - Impact of interventions in all districts in Tanzania, - Impact of current NMSP strategies, - Evaluation of NMSP target and suggestion of aggressive intervention to reach such optimistic target. | - Impact of potential strategies, - Predicted costs per strata per intervention of the strategies in the revised NMSP, - Differences in impact and in costs of current and revised NMSP. | - Historical trend and baseline prevalence fitted to estimates from new prevalence risk map, - Updated predicted impact and costs of interventions and comparison to previous outcomes. |

ALMA= The African Leaders Malaria Alliance, CHAI = Clinton Health Access Initiative, IHI= Ifakara Health Institute, MoHCDGEC = Ministry of Health, Community Development, Gender, Elderly and Children; NIMR= National Institute for Medical Research, NBS = National Bureau of Statistics, MUHAS= Muhimbili University of Health and Allied Sciences, PMI = President Malaria Initiative, TMA = Tanzania Meteorological Agency, UDSM= University of Dar es Salaam, Swiss TPH = Swiss Tropical and Public Health Institute, RTI = Research Triangle Institute, WHO = World Health Organisation
