# Supplementary figures and images for: Applied mathematical modelling to inform national malaria policies, strategies and operations in Tanzania
Source: Malar J. 2020 Mar 2;19:101. doi: 10.1186/s12936-020-03173-0 (PMC7053121; doi:10.1186/s12936-020-03173-0)

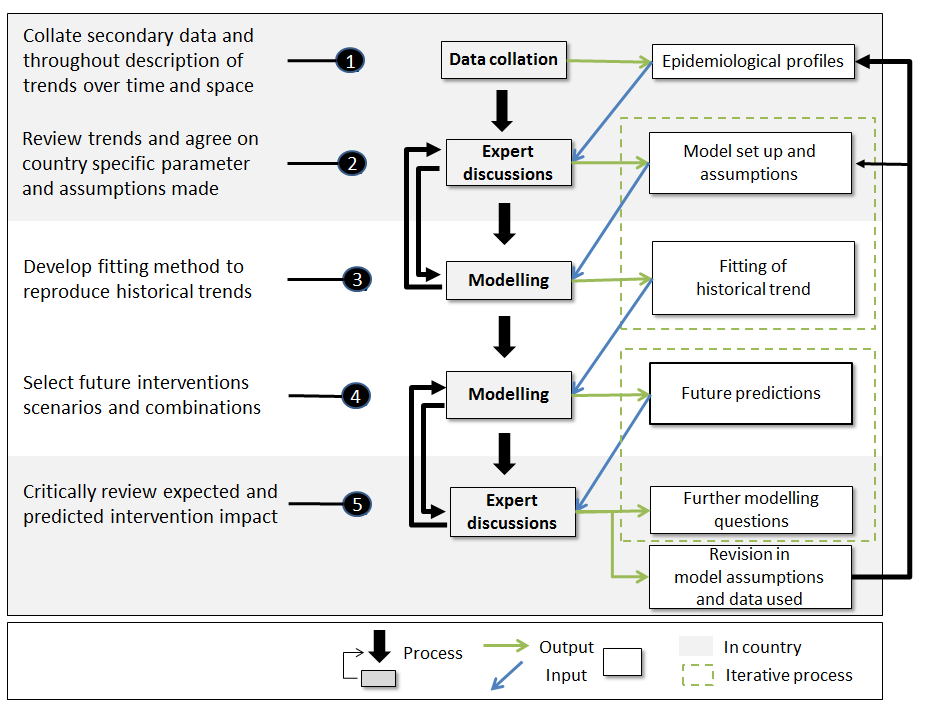

Supplement: Supplementary file 2 — Additional file 2. Iterative process between modelling and in-country discussions [file 12936_2020_3173_MOESM2_ESM.tif]
